# Investigation of the Structural, Thermal, and Physicochemical Properties of Nanocelluloses Extracted From Bamboo Shoot Processing Byproducts

**DOI:** 10.3389/fchem.2022.922437

**Published:** 2022-06-14

**Authors:** Tong Lin, Qi Wang, Xuan Zheng, Yu Chang, Hui Cao, Yafeng Zheng

**Affiliations:** ^1^ College of Food Science, Fujian Agriculture and Forestry University, Fuzhou, China; ^2^ Institute of Agricultural Engineering, Fujian Academy of Agriculture Sciences, Fuzhou, China; ^3^ Fujian Key Laboratory of Agricultural Product (Food) Processing, Fuzhou, China; ^4^ Institute of Chinese Medical Sciences, University of Macau, Macau SAR, China; ^5^ Faculty of Food Science and Technology, University of Vigo, Pontevedra, Spain

**Keywords:** nanofibrillated cellulose, nanocrystalline cellulose, bamboo shoot, acid hydrolysis, physicochemical property

## Abstract

Nanocellulose has gained increasing interest due to its excellent properties and great potential as a functional component or carrier in food and pharmaceutical industries. This study investigated the structural, thermal, and physicochemical properties of nanofibrillated cellulose (NFC) and nanocrystalline cellulose (CNC) extracted from bamboo shoot (*Leleba oldhami* Nakal) processing byproducts. NFCs were prepared through low concentration acid hydrolysis combined with ultrasonic treatment. CNCs were further isolated from NFCs using sulfuric acid hydrolysis treatment. TEM images showed that NFC and CNC exhibited typical long-chain and needle-like structures, respectively. CNC suspension was stable due to its zeta potential of -34.3 ± 1.23 mV. As expected, both NFC and CNC displayed high crystallinity indexes of 68.51 and 78.87%, and FTIR analysis confirmed the successful removal of lignin and hemicellulose during the treatments. However, the thermogravimetric analysis indicated that sulfuric acid hydrolysis decreased the thermal stability of CNCs. The improved physicochemical properties of NFC and CNC suggested their potential in various applications.

## 1 Introduction

Cellulose, the main component of plant cell walls, is the most abundant biomaterial in the world with its biodegradability and renewability (Eichhorn et al., 2010). Recently, to increase the value of cellulose-rich processing residues, effective conversion of cellulose from various plant-derived residues into nanocellulose, mainly nanofibrillated cellulose (NFC) or cellulose nanocrystals (CNCs), has received increasing attention due to their great potential on biomedical and food applications (Kaushik and Singh 2011; Pelissari et al., 2013; Chen et al., 2016; Perumal et al., 2022; Sainorudin et al., 2022). NFC is a novel nanomaterial with soft and long-chain structures (typical diameter of 4–20 nm and length of 500–2000 nm) with a high aspect ratio, which is produced based on a fibrillation process (Yan et al., 2018). CNC is a spherical “rod” or “needle” with a highly crystalline structure with a diameter of 3–10 nm and a length of 100–1,000 nm (Marett et al., 2017). The physical dimensions and related properties of nanocellulose could be varied based on the source of cellulose and the applied preparation conditions (Mishra et al., 2019; Holilah et al., 2022).

In recent years nanocellulose has attracted intensive research attention and been used in many industrial applications, which can be attributed to its special properties, including high specific strength and stiffness, high reinforcing potential, large specific surface area, and biocompatibility (Ghaderi et al., 2014; Reshmy et al., 2020; Almashhadani et al., 2022). Moreover, non-toxic nanocellulose also exhibits promising potential in the food industry, such as biodegradable food packaging, novel delivery systems, food stabilizer, and functional food ingredients (Mu et al., 2019). It was reported that the rising demand and the novel applications of nanocellulose have driven the industry to build a number of new manufacturing facilities (Mariano et al., 2014; Coelho et al., 2018).

Bamboo shoots have been traditionally consumed as delicious forest vegetables and healthcare food worldwide, especially in Asian countries (Choudhury et al., 2012). As fresh bamboo shoots have short shell life, most of them are dried, canned, or fermented into various food products for consumption during off-seasons (Satya et al., 2010; Choudhury et al., 2012). However, only the tender top part (around 50% w/w) of bamboo shoots can be utilized; therefore, the fast-growing bamboo shoot processing industry in China creates a huge amount of processing byproducts. Some research achievements have been made on the innovative utilization of bamboo shoot byproducts (Zhang et al., 2017a; Zhang et al., 2017b), but most of those still have been discarded directly resulting in waste of resources and environmental problems (Chen et al., 2018; Chen et al., 2019). Bamboo shoot processing byproducts consist of a considerable amount of cellulose (Peng and She 2014), representing an abundant and cheap source for production of nanocellulose, such as NFC or CNC based on different application purposes. To increase the marketability of nanocellulose, numerous agricultural or processing byproducts, such as banana peel (Pelissari et al., 2013; Khawas and Deka 2016), cassava bagasse (Travalini et al., 2017), cotton stalks (Soni et al., 2015), and rice husk (Johar et al., 2012), have been investigated as new sources for the nanocellulose production. In a previous study, nanocrystalline cellulose with a length of 200–500 nm and a diameter less than 20 nm was prepared from bamboo pulp through chemical treatment (Yu et al., 2012). However, to the best of our knowledge, bamboo shoot (*Leleba oldhami* Nakal) processing byproducts have not been investigated as a source for NFC and CNC production.

In this study, bamboo shoot NFC was prepared through low concentration acid hydrolysis combined with ultrasonic treatment, which is a relatively green method with the reduced use of chemical reagents. Thereafter, the NFC is subjected to sulfuric acid hydrolysis to further remove the amorphous region in the fiber to obtain CNC. Multiple analysis methods were applied to determine the morphological, structural, and thermal properties of the as-prepared samples. Moreover, to estimate their potential in the food application, the physicochemical properties, including water/oil binding and swell capacities were investigated and compared.

## 2 Materials and Methods

### 2.1 Materials

Bamboo shoot (*Leleba oldhami* Nakal) processing byproducts (basal parts of the bamboo shoot) were obtained from a local bamboo shoot processing company in Fujian province, China. Sulfuric acid, hydrochloric acid, ethyl acetate, and hydrogen peroxide were purchased from Fuyu Fine Chemical Co., Ltd. (Tianjin, China). Sodium hydroxide (tablet) and anhydrous ethanol were purchased from Wokai Biotechnology Co., Ltd. (Shanghai, China). All other chemicals were of analytical grade.

### 2.2 Isolation of Insoluble Fiber

The isolation procedure of bamboo shoot insoluble dietary fiber (IDF) was adapted from Luo et al. (2018) with modifications. The bamboo shoot byproducts were cut into small pieces (3–5 cm), dried, and ground into fine powder. The resulting powder was soaked in ethyl acetate at a ratio of 1:4 (w/v) for 1 h and then washed with distilled water to obtain a degreased sample. Then, it was added to distilled water at a ratio of 1:30 (w/v), leached at 80°C for 2 h, then washed with 78% ethanol, and dried for 24 h to obtain IDF (100 mesh).

### 2.3 Preparation of NFC

The preparation of NFC from IDF was carried out according to the reported methods with modifications (Kaushik and Singh 2011; Leite et al., 2017). IDF powder was pretreated with 5% (w/v) NaOH solution at a ratio of 1:20 (w/v) at 50°C, and the suspension was stirred for 4 h. The mixture was centrifuged to achieve the residue, which was then washed with distilled water. The procedure was repeated until the fiber was free of alkali. To remove the residual lignin and hemicellulose, the alkali-treated fiber was then bleached with 8% (v/v) hydrogen peroxide (H_2_O_2_) at room temperature for 24 h, then washed and centrifuged (2 times, 10,000 rpm/15°C/10 min). The bleached fiber was subjected to low concentration acid hydrolysis combined with ultrasonic treatment to isolate NFC. The fiber was mixed with 5% (v/v) HCl solution at a ratio of 1:30 (w/v), and treated with an ultrasonic transducer (KQ2200DE, Ultrasonic Instrument, Kunshan, China) at the ultrasonic power of 170 W at 56°C for 80 min. The procedure was repeated under the same conditions except for the increased HCl concentration (10%, v/v). The resulting mixture was neutralized with NaOH and washed with distilled water. The precipitate was freeze-dried to obtain NFC powders.

### 2.4 Preparation of CNC

The sulfuric acid hydrolysis was conducted on NFC powders to prepare CNC (Johar et al., 2012). Briefly, NFC powder was mixed with a preheated sulfuric acid solution (62%, w/v) at a ratio of 1:20 (w/v) at 37°C for 80 min under continuous stirring of 600 rpm. The hydrolyzed material was washed and centrifuged (4,000 rpm, 10 min) several times to remove residual sulfuric acid. The suspension was dialyzed against distilled water and homogenized for 5 min. The suspension was lyophilized to obtain CNC, and kept refrigerated for further analysis.

### 2.5 Surface Morphology Analysis

To observe the surface morphology and determine the dimensions of NFC and CNC, 1.0 mg of sample powder was dispersed in 10 ml of deionized water and sonicated for 15 min to disperse the agglomerated fibers. After sonication, a drop of dispersion was deposited on the carbon-coated copper grid and allowed to dry at room temperature. Transmission electron microscope (TEM) images were obtained under an acceleration voltage of 200 kV using a transmission electron microscope (THS-117, Tecnai, United States). The dimension and aspect ratio of samples were measured according to the TEM images using the software (Nano Measurer 1.2).

### 2.6 Zeta Potential Analysis

The samples were diluted with deionized water at a fiber/liquid ratio of 1:5. The surface charge (zeta potential) of diluted fiber suspensions was measured at 25°C using a Zetasizer Nano ZS90 analyzer (Malvern, United Kingdom).

### 2.7 Fourier Transform Infrared Spectroscopy

To analyze and compare the functional groups of nanocellulose samples, each sample was finely ground and mixed with KBr. The mixture was then pressed into a transparent film and analyzed by a Fourier transform infrared spectroscopy (Vertex 70/70 v, Bruker, Germany). FTIR spectra were recorded in a measurement range of 300–4,000 cm^−1^ with a resolution of 4 cm^−1^.

### 2.8 X-Ray Diffraction

To examine the crystallinity of the fiber samples, XRD images were scanned in the range 2θ = 5–50° and recorded at a speed of 5°/min using an X-ray diffractometer (D8 Rigaku9000, BrukerAXS, Karlsruhe, Germany). The crystallinity index (CrI) is calculated according to Eq. 1.

$$\text{CrI}\left(\text{\%}\right)=\left(1-\frac{I_{\left(am\right)}}{I_{\left(200\right)}}\right)\times 100\text{\%}$$ (1)

where I_(am)_ is the diffraction intensity of the only amorphous regions at around 2θ = 18°, and I_(200)_ represents the maximum diffraction intensity of both crystalline and amorphous regions at around 2θ = 22.6° (Travalini et al., 2017).

### 2.9 Thermogravimetric Analysis

The dried samples (5.0 mg) were subjected to thermal stability analysis using a thermogravimetric analyzer (4F3, NETZSCH, Germany). The mass change in the range of 30–790°C was measured at a constant heating rate of 10°C/min under a nitrogen atmosphere, and the sample was kept at 790°C for 5 min.

### 2.10 Physicochemical Properties

#### 2.10.1 Swelling Capacity

Accurately weigh 0.5 g of sample (W_d_) in a 10 ml calibrated tube and its initial bed volume was recorded as V_1_, and mixed with 5.0 ml of distilled water and shaken well. The tube was placed in a 25°C water bath for 24 h, and the bed volume of fiber in the liquid was carefully measured and recorded as V_2_. SC was calculated according to Eq. 2:

$$\mathrm{SC}\left(\mathrm{mL}/g\right)=\frac{V_{2}-V_{1}}{W_{d}}$$ (2)

### 2.11 Water Holding Capacity

The dried sample (0.5 g, M_d_) was transferred into a 50 ml centrifuge tube, mixed with 25 ml of deionized water, and then placed in a 37°C water bath for 30 min with continuous stirring. The mixture was centrifuged at 4,000 rpm for 15 min, and the supernatant was discarded. The precipitated wet powder (M_w_) was collected and weighed. WHC was calculated according to Eq. 3:

$$\mathrm{WHC}\left(g/g\right)\frac{M_{w}-M_{d}}{M_{d}}$$ (3)

### 2.12 Oil Binding Capacity

The dried sample (1.0 g, M_1_) was mixed with 20 ml of edible blend oil in a 50 ml centrifuge tube and stirred for 30 s every 5 min. After 1 h, the tube was centrifuged at 4,000 rpm for 15 min, and the upper layer of oil was decanted. The residue (M_2_) was blotted with filter paper to dry the free oil and weighed. OBC was calculated according to Eq. 4:

$$\mathrm{OBC}\left(g/g\right)\frac{M_{2}-M_{1}}{M_{1}}$$ (4)

### 2.13 Statistical Analyses

All results are expressed as mean ± SD. *p* values less than 0.05 were considered to indicate a significant difference between treatments. Data were analyzed using SPSS/11.5 software.

## 3 Results and Discussion

### 3.1 Morphology, Dimension and Surface Charge

**FIGURE 1:**
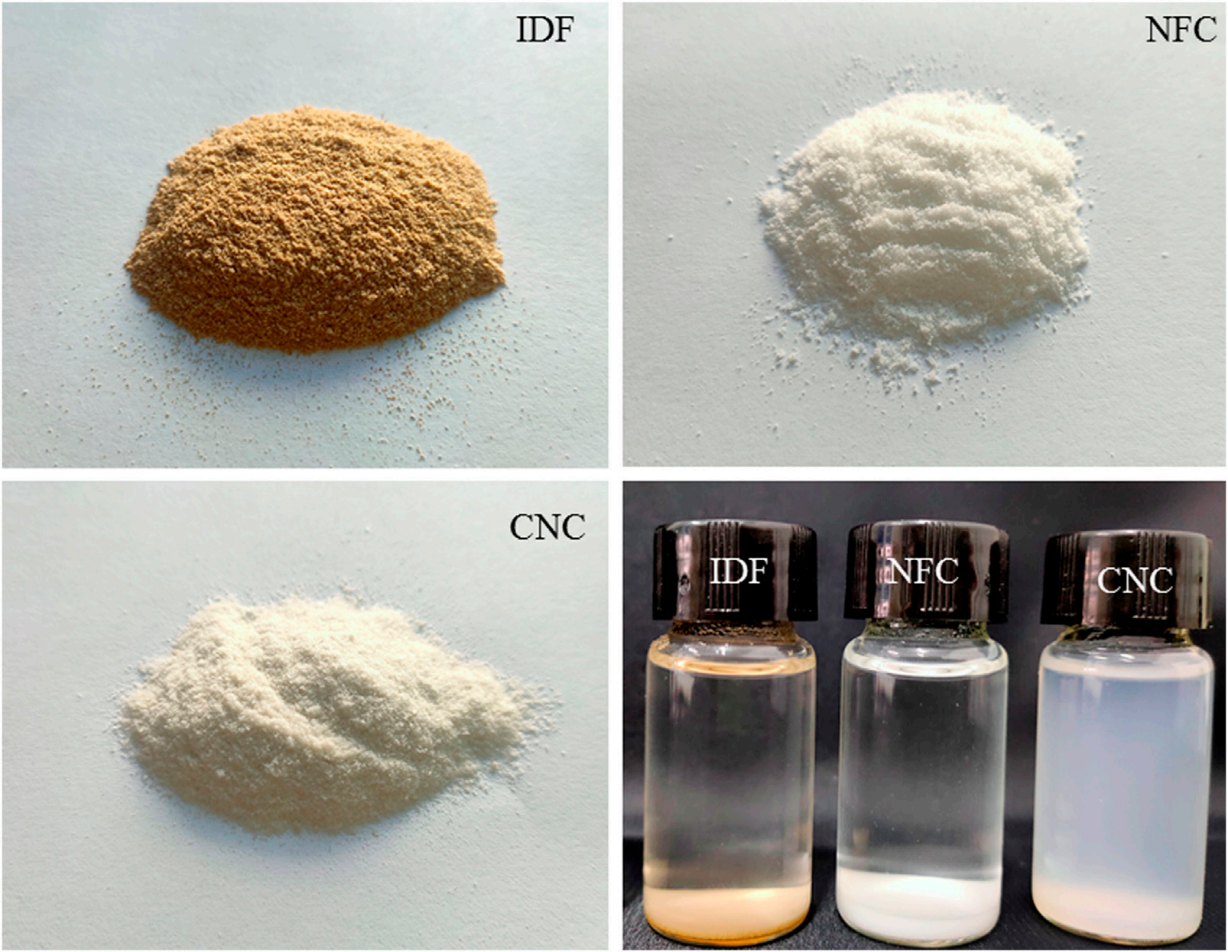
Photographs of IDF, NFC, and CNC powders and their aqueous suspensions after setting aside at room temperature for 24 h.

The images of the IDF, NFC, and CNC isolated from bamboo shoot processing byproducts and their suspensions are shown in Figure 1. The color of the bamboo shoot IDF is brown due to the presence of non-cellulosic materials and other impurities, including main lignin, hemicelluloses, and pectin (Johar et al., 2012). Obviously different from IDF, the color of NFC and CNC powders appear completely white, indicating the presence of almost pure cellulosic material and the successful removal of non-cellulosic materials and other impurities during the chemical and physical treatments. The dispersibility of the samples was observed by dispersing the powder (50 mg) in 10 ml of water and setting it aside at room temperature for 24 h. Compared to IDF and NFC, CNC exhibited a significantly stronger ability to disperse in water and provided a more stable suspension. One of the main reasons was due to the sulfuric acid hydrolysis used for the isolation of CNC. As the most popular and effective method for CNC production, sulfuric acid is able to react with the surface hydroxyl groups to form negatively charged sulfate ester groups randomly distributed on the surface of CNC, promoting their dispersion in water (Mariano et al., 2014).

**FIGURE 2:**
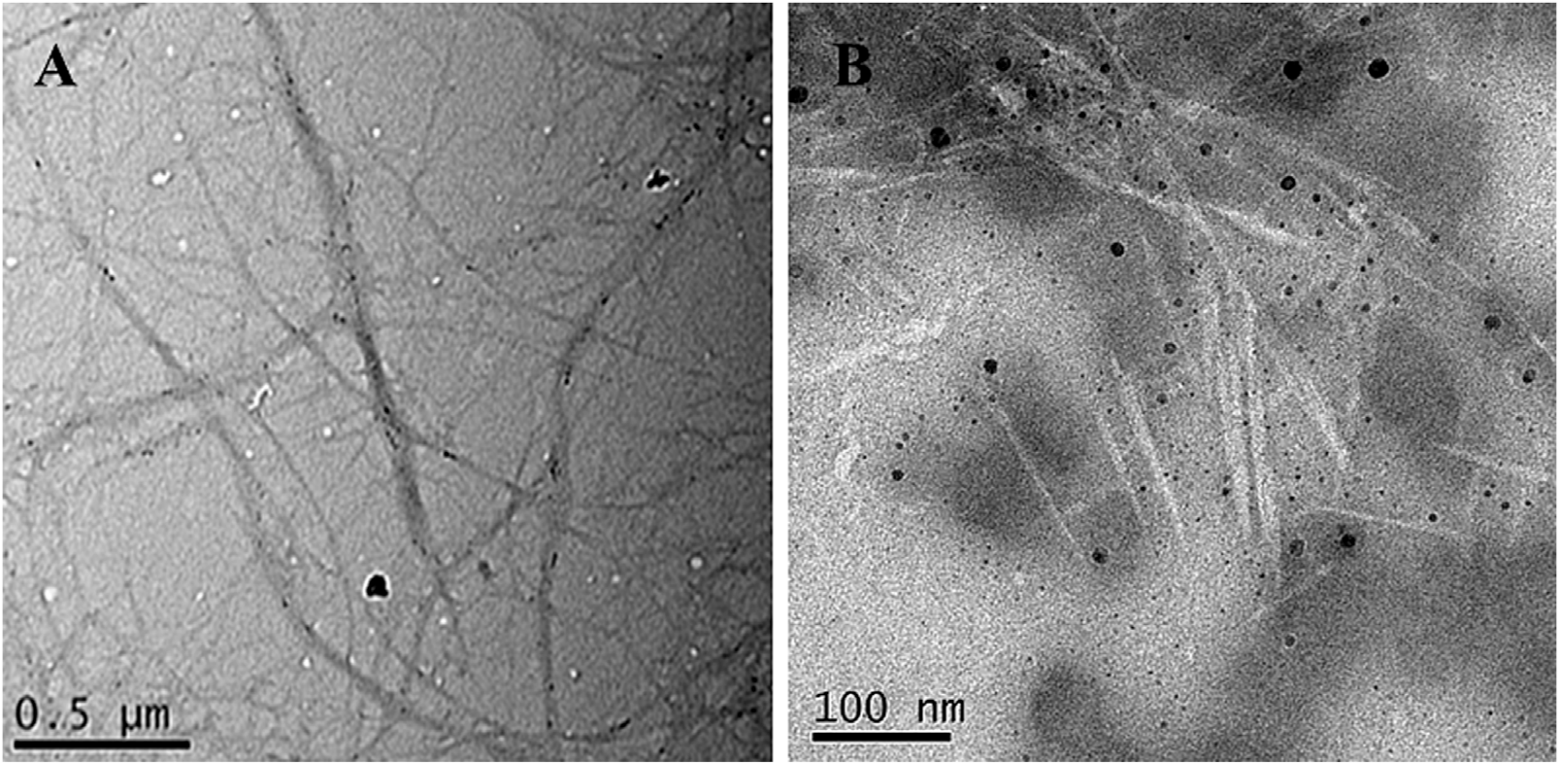
TEM images of bamboo shoot NFC **(A)** and CNC **(B)**.

As shown in Figure 2, after pretreatment and low concentration acid hydrolysis combined with ultrasonic treatment, raw insoluble fiber was broken and crushed into individualized NFCs with a soft and long-chain structure. NFC was then subjected to sulfuric acid hydrolysis, which further destroyed the long-chain structure of NFCs into the typical needle-like structure of CNC. The results from Table 1 revealed that the average length and diameter of NFC were 952.45 ± 38.53 nm and 69.97 ± 3.21 nm, respectively. Thus, its aspect ratio was 13.64 ± 1.61. It is notable that the width of NFC is higher than the nanofibrillated cellulose obtained in other studies (Winuprasith and Suphantharika 2013; Yan et al., 2018), suggesting that the method applied in this study was relatively green but also less efficient due to low concentration acid applied. The diameter and length of CNC were 7.23 ± 2.07 nm and 124.50 ± 51.94 nm, with an aspect ratio of 18.19 ± 2.45. Bamboo shoot CNC with a high aspect ratio is useful to enhance and improve the mechanical properties of composite materials under low loads (Liu et al., 2017).

**TABLE 1:** Dimensions and zeta potential of NFC and CNC isolated from bamboo shoot processing byproducts.

Sample	Length (nm)	Diameter (nm)	Aspect Ratio	Zeta Potential (mV)
NFC	952.45 ± 38.53^a^	69.97 ± 3.21^a^	13.64 ± 1.61^b^	-15.5 ± 0.87^a^
CNC	124.50 ± 51.94^b^	7.23 ± 2.07^b^	18.19 ± 2.45^a^	-34.3 ± 1.23^b^

^a,b^ Different letter superscripts in the same column indicate a statistically significant difference (p < 0.05).

### 3.2 Zeta Potential Analysis

As shown in Table 1, both NFC and CNC aqueous suspensions exhibited negative zeta potential (surface charge), and CNC presented a significantly lower zeta potential value of −34.3 ± 1.23 mV than that of NFC (−15.5 ± 0.87 mV). Electrostatic repulsion of the CNC suspension was enhanced due to the connection of negatively charged sulfate groups to the nanofiber surface during the sulfuric acid hydrolysis. It has been reported that if the absolute value of nanofiber’s zeta potential is higher than 30, the nanofiber aggregation will be greatly minimized, which could be desirable for many applications (Leite et al., 2017).

### 3.3 X-Ray Diffraction

**FIGURE 3:**
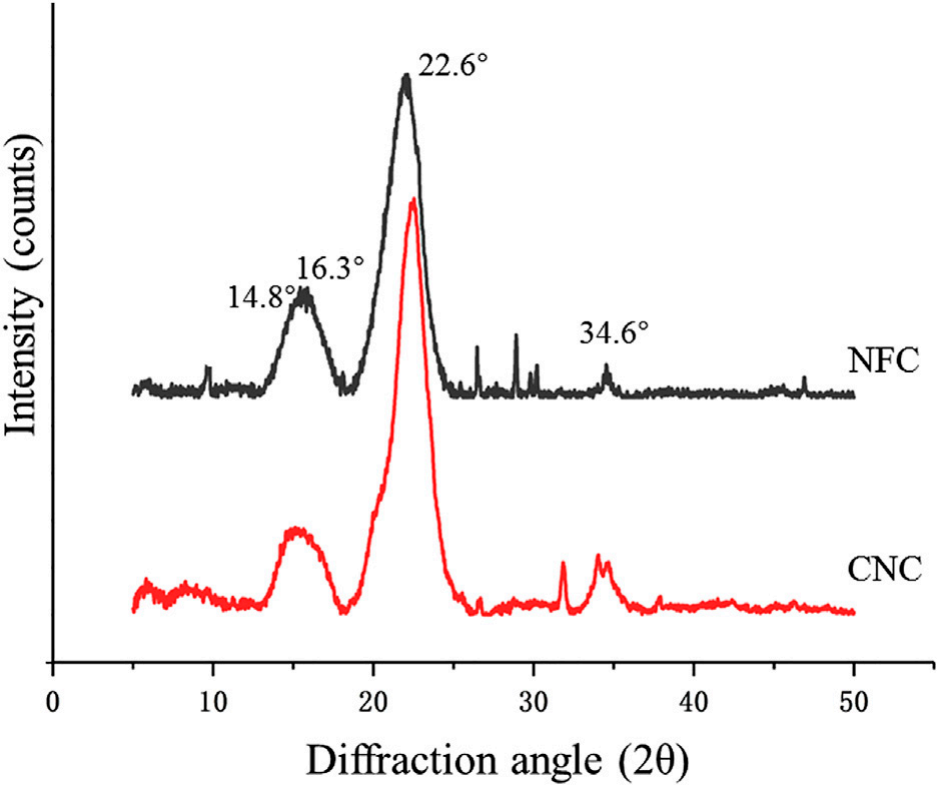
X-ray diffraction pattern of bamboo shoot NFC and CNC.

X-ray diffraction (XRD) analysis was used to evaluate XRD patterns and changes in the crystallinity of bamboo shoot NFC and CNC (Figure 3). It was noticed that both NFC and CNC had diffraction peaks at 2θ = 14.8°, 16.3°, 22.6°, and 34.6°, indicating that the cellulose was in the form of cellulose I (Marett et al., 2017). The peak position of the nanocelluloses was not obviously different, suggesting that the crystalline structure of CNC did not change after subjecting NFC to sulfuric acid hydrolysis. Meanwhile, the diffraction peak of CNC at 22.6° was sharper than that of NFC, and the intensity of the diffraction peak of the amorphous region is significantly decreased, indicating an increase in crystallinity. The calculated crystallinity indexes of NFC and CNC were 68.51 and 78.87%, respectively. The high crystallinity was strongly related to the different treatments of fiber samples. NFC was prepared from IDF through low concentration acid hydrolysis combined with ultrasonic treatment, which effectively destroyed the amorphous lignin and hemicellulose to release the nanofibrillated cellulose. Sulfuric acid hydrolysis further dissolved the remained amorphous regions (mainly hemicellulose) in NFC, releasing more individual needle-like crystallites by promoting the hydrolytic cleavage of glycosidic bonds. The crystallinity of CNC prepared from bamboo shoot residue was higher than that of cellulose nanocrystalline isolated from the bamboo pulp (Yu et al., 2012) or other agricultural wastes (Liu et al., 2017; Rohaizu and Wanrosli 2017). Therefore, CNC prepared in this study could provide promising reinforcement potential for promising the composite materials (Marett et al., 2017).

### 3.4 Fourier Transform Infrared Spectroscopy

**FIGURE 4:**
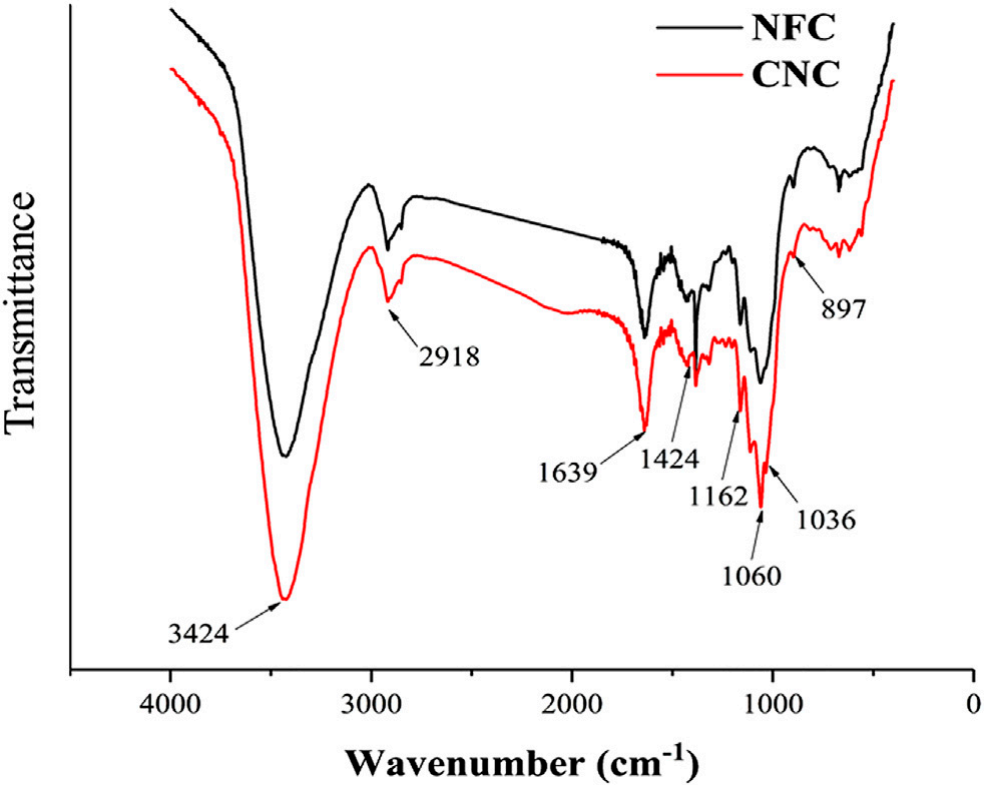
FTIR spectra of bamboo shoot NFC and CNC.

The FTIR spectra of NFC and CNC (Figure 4) could be used to provide direct information on chemical changes that occur during the different treatments for the isolation of NFC and CNC from bamboo shoot byproducts. According to the literature, there are several characteristic bands related to the chemical structure of the non-cellulosic materials, mainly lignin and hemicellulose. The bands between 1,600 and 1,500 cm^−1^ correspond to the vibrations of the aromatic rings of lignin. The peak at 1730 cm^−1^ in the FTIR spectrum is attributed to the ester groups in hemicellulose or carboxylic group in the ferulic and *p*-coumaric acids of lignin (Mandal and Chakrabarty 2011). Likewise, the peak at around 1,240 cm^−1^ stand for the elongation of the ether linkages in the lignin (Travalini et al., 2017). All these mentioned bands have disappeared in the FTIR spectra of NFC and CNC, meaning the successful removal of non-cellulosic material upon the treatments.

The main absorption peak located near 3,424 cm^−1^ in the spectrum is formed by the free O-H stretching vibrations of the OH groups in the cellulose molecule (Alemdar and Sain 2008; Johnson et al., 2011). Moreover, the absorption peaks at 2,918 cm^−1^, 1,639 cm^−1,^ and 1,424 cm^−1^ are attributed to the characteristic C-H stretching vibration, the O-H bending of the adsorbed water, and the CH_2_ bending vibration of the crystalline region in cellulose (Mandal and Chakrabarty 2011; Wang et al., 2017). The absorption peak at 1,162 cm^−1^ is derived from the stretching of the glycosidic bond anti-symmetric C-O-C (Pereira et al., 2017). In both samples, the observed peaks at 1,060 and 897 cm^−1^ refer to the C-O vibration and C-H stretching in the cellulose chains (Johar et al., 2012). These results indicate that the cellulose component was not destroyed or removed after the chemical treatments carried out on the bamboo shoot residues and remained as dominating material presented in both samples, which is consistent with the result from the XRD analysis. Additionally, the absorption peaks at 1,060 and 1,036 cm^−1^ of CNC were shapers than those of NFC, which could be due to that the C-O bond stretching of CNC was affected by the sulfonic acid groups after the sulfuric acid hydrolysis (Li et al., 2018).

### 3.5 Thermogravimetric Analysis

**FIGURE 5:**
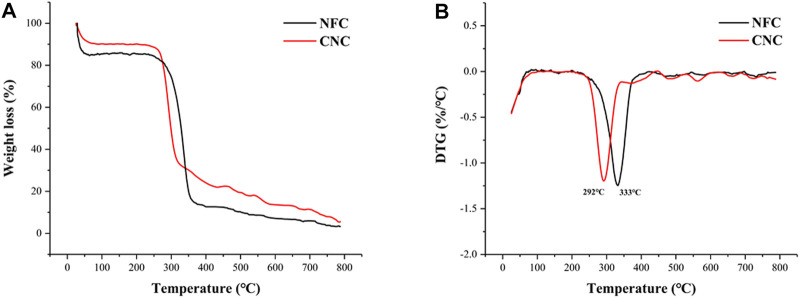
TG **(A)** and DTG **(B)** curves of bamboo shoot CNC and NFC.

Thermogravimetric (TG) and derivative thermogravimetric (DTG) curves obtained for NFC and CNC are shown in Figure 5. It was observed that both NFC and CNC had two decomposition stages. The initial weight loss starting at 30°C and continuing up to around 100°C could be attributed to the evaporation of loosely bound moisture on the fiber surface and the intermolecularly H-bonded water (Khawas et al., 2016), which had been confirmed by the characteristic peak of FTIR spectra at 1,639 cm^−1^. In the next stage, the sharp decomposition peaks at around 300°C were noticed and corresponded to cellulose decomposition (Kaushik and Singh 2011). Meanwhile, it was notable that there were no characteristic peaks attributing to the thermal decomposition of lignin and hemicellulose were found, which was consistent with the XRD and FTIR results. In the second decomposition stage, NFC started to degrade at around 235°C, and the rate of degradation reached a maximum at 333°C. However, obviously different from the degradation behavior of NFC, CNC showed lower start and main degradation temperatures at around 224 and 292°C, respectively. The decreased thermal stability of CNC might be corresponded to its high surface area to volume ratio and the introduced sulfated groups in the cellulose crystals (Mandal and Chakrabarty 2011; Lu et al., 2013). Compared to NFC, a greater surface area of CNC exposed to heat contributes to diminishing its thermal stability. Moreover, the highly sulfated regions presented on the surface of cellulose crystals after sulfuric acid hydrolysis are more accessible and degradable. As shown in the TG curve, CNC showed a slower weight loss rate than that of NFC after the temperature of 350°C, which could be explained by the fact that the unsulfated crystal interior of CNC is dense and compact. Subsequent slow decomposition at the temperature range of 400–530°C corresponds to the oxidative decomposition of carbon (Nada and Hassan 2000).

### 3.6 Physicochemical Properties

**FIGURE 6:**
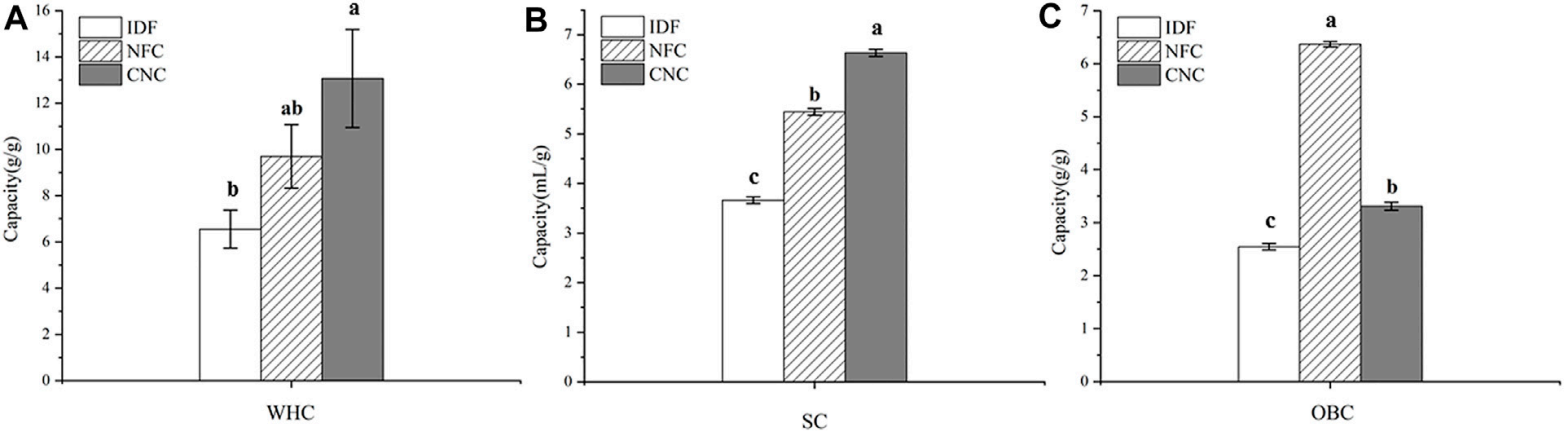
Physicochemical properties of bamboo shoot IDF, NFC, and CNC. **(A)** Water holding capacity, **(B)** swelling capacity, and **(C)** oil holding capacity.

Hydration properties, including swelling capacity (SC) and water holding capacity (WHC), are one of the important factors influencing the success of the incorporation of fiber ingredients into foods requiring moisture retention. As shown in Figure 6, after different chemical and physical treatments, SC values of NFC (5.44 ml/g) and CNC (6.63 ml/g) were significantly (*p* < 0.05) increased by 48.6 and 81.1%, compared with that of IDF (3.66 ml/g). Similarly, WHC values of NFC (9.70 g/g) and CNC (13.07 g/g) also significantly (*p* < 0.05) increased by 48.1 and 99.5%, compared with that of IDF (6.55 g/g). The improved hydration properties of NFC and CNC could be explained by the decreased particle size after the removal of lignin and hemicellulose, and therefore the increased surface area provides more binding sites for water molecules. Moreover, the broken cellulose chain structure in NFC and CNC also contributed to the increased space-enlarging effects and thus swelling capacity (Lu et al., 2013). The sulfated regions in the surface of CNC might also provide enhanced interchain spaces for the entrapment of moisture, which further contribute to its hydration properties (Mandal and Chakrabarty 2011).

OBC is an important factor for the dietary fibers to adsorb or bind the lipid components and thus be appropriate for the stabilization of foods with a high content of fat, and emulsions. Bamboo shoot dietary fiber has been proven to be effective on oil binding, which contributes to its weight control function (Park and Jhon 2009; Luo et al., 2017). In this study, IDF extracted from bamboo shoot residue exhibited a promising OBC value of 2.55 g/g, which is significantly higher than that of fruit dietary fibers ranging from 0.7 to 1.6 g/g (Martinez et al., 2012). After low concentration acid hydrolysis combined with ultrasonic treatment, the OBC of NFC significantly increased by 2.5-fold–6.37 g/g (*p* < 0.05). Considering its greater surface area, CNC prepared from NFC by sulfuric acid hydrolysis is expected to exhibit a great capacity to bind the oil molecules. However, CNC showed a decreased OBC (3.31 g/g), compared with that NFC. This opposite result might be related to the fact that a large amount of CNC powder suspended in the upper layer of oil could not be precipitated, thus greatly lowering the OBC value.

## 4 Conclusion

This study investigated the morphological, crystal, thermal, and physicochemical properties of bamboo shoot NFC and CNC. CNC had a high-zeta potential of −34.3 mV, contributing to its suspension stability. The crystal type of NFC and CNC retained a type of cellulose I pattern with the crystallinity indexes of 68.51 and 78.87%, respectively. FTIR spectra evidenced about the successful removal of lignin and hemicellulose during the treatments. Thermogravimetric analysis indicated that CNC had lower thermal stability than that of NFC, which might be corresponded to its high surface area and the introduced sulfated groups in the cellulose crystals. NFC and CNC exhibited promising physicochemical properties, indicating that they are suitable to be incorporated into various foods with different purposes. However, a thorough toxicity assessment of these novel nanocelluloses needs to be carried out before their applications in the food industry.

## Data Availability

The original contributions presented in the study are included in the article/Supplementary Material; further inquiries can be directed to the corresponding author.
